# Lipopolysaccharide-induced inflammatory modulation of the apoptotic pathways in human primary aniridia limbal stromal cells, in vitro

**DOI:** 10.1371/journal.pone.0361130

**Published:** 2026-10-27

**Authors:** Shanhe Liu, Shuailin Li, Shao-Lun Hsu, Fabian N. Fries, Zhen Li, Swarnali Kundu, Berthold Seitz, Maryam Amini, Shweta Suiwal, Julia Zimmermann, Simon Trusen, Tanja Stachon, Nóra Szentmáry

**Affiliations:** 1 Dr. Rolf M. Schwiete Center for Limbal Stem Cell and Congenital Aniridia Research, Saarland University, Homburg/Saar, Germany; 2 Department of Experimental Ophthalmology, Saarland University, Homburg/Saar, Germany; 3 Department of Ophthalmology, Saarland University Medical Center, Homburg/Saar, Germany; Marshall University, UNITED STATES OF AMERICA

## Abstract

**Purpose:**

This study investigated apoptosis-related responses to a standardized 24-hour lipopolysaccharide (LPS) challenge in primary limbal stromal cells (LSCs) derived from patients with congenital aniridia (AN-LSCs) and control donors.

**Methods:**

LSCs were isolated from limbal tissue obtained from patients with congenital aniridia and control donors. Cells were treated with 0, 2.5, 10, and 17.5 µg/mL LPS for 24 hours. Apoptosis was assessed using Annexin V-APC/PI staining and flow cytometry. Apoptosis-related mRNA levels were quantified by qPCR, cell-associated protein fluorescence was assessed by flow cytometry, and secreted TNF-α was measured by ELISA.

**Results:**

The mean basal apoptosis rate was numerically higher in AN-LSCs than in control LSCs (2.10% vs. 0.52%), although the donor-level difference was not statistically significant. The exploratory linear mixed-effects model showed a significant main effect of LPS condition and a significant Group × LPS interaction. Apoptosis increased significantly at 10 and 17.5 µg/mL LPS compared with the corresponding untreated condition in both groups, and the increase from the untreated condition to 17.5 µg/mL LPS was significantly greater in AN-LSCs than in control LSCs (adjusted P = 0.003). qPCR and flow-cytometry analyses identified marker-specific LPS-associated changes, but no molecular Group × LPS interaction reached statistical significance. Cell-associated TNF-α fluorescence was distinguished from secreted TNF-α measured by ELISA.

**Conclusions:**

Acute LPS exposure increased apoptosis in both groups, with a greater increase in AN-LSCs at the highest concentration. Molecular changes were marker- and concentration-specific without evidence of differential molecular response patterns between groups. Given the small number of independent donors, the marked donor-age imbalance, and the acute nature of the model, these findings should be considered exploratory and hypothesis-generating and require confirmation in a larger independent cohort of appropriately age-matched donors.

## Introduction

Congenital aniridia is a rare panocular developmental disorder primarily caused by *PAX6* haploinsufficiency, resulting in iris hypoplasia and a spectrum of ocular malformations [1–3]. A defining complication of aniridia is aniridia-associated keratopathy (AAK), a progressive, degenerative condition characterized by limbal stem cell deficiency (LSCD). In AAK, the depletion of limbal epithelial stem cells and disruption of their niche function lead to chronic epithelial breakdown, corneal neovascularization, conjunctivalization, and ultimately, corneal scarring with significant vision impairment [1,4]. This LSCD is thought to stem from a dysfunctional limbal niche, which encompasses not only the epithelial stem cells but also their supportive stromal microenvironment [5,6].

Limbal stromal cells (LSCs), located within the limbal niche, are critical for maintaining corneal integrity and homeostasis. These mesenchymal cells contribute to the support of limbal epithelial stem cells and facilitate corneal epithelial renewal through paracrine trophic signaling and extracellular matrix remodeling. In a healthy state, LSCs help preserve corneal transparency and immune privilege by actively suppressing inflammation and fibrosis [7]. Disruption of this stromal support amplifies ocular surface pathology; notably, *PAX6* deficiency has been associated with impaired wound healing in aniridia, characterized by heightened inflammation and increased stromal cell apoptosis, ultimately contributing to corneal opacity [5,8]. These findings underscore the pivotal role of the limbal stroma in orchestrating both regenerative and inflammatory responses within the corneal microenvironment.

Emerging evidence indicates systemic dysregulation of apoptosis in aniridia. Proteomic analyses of conjunctival specimens from affected individuals have revealed significant alterations in components of the apoptotic pathway [9]. Complementary studies have also identified dysregulated expression of apoptosis-related microRNAs within the limbal stromal niche of aniridia patients [10]. Despite these insights, the intrinsic apoptotic signaling mechanisms in primary human aniridic limbal stromal cells (AN-LSCs) remain poorly understood—particularly in the context of inflammatory stress.

Inflammation is a well-established inducer of apoptosis in ocular surface disorders. Lipopolysaccharide (LPS), a component of the outer membrane of Gram-negative bacteria, is commonly used as a standardized acute innate inflammatory stimulus. Through activation of Toll-like receptor 4 (TLR4) signaling, LPS induces downstream innate immune responses and has been associated with apoptosis in experimental models of corneal injury [11–13]. The present model was designed to compare short-term cellular responses to a standardized acute LPS challenge in control and aniridia-derived limbal stromal cells rather than to reproduce the complex chronic inflammatory microenvironment of AAK. Apoptosis at the ocular surface is primarily executed by caspase proteases, which orchestrate the dismantling of cellular components [14], and is tightly regulated by a network of pro- and anti-apoptotic factors. Mitochondrial apoptotic signaling is governed by the BCL-2 protein family, where the balance between anti-apoptotic members (e.g., BCL-2) and pro-apoptotic members (e.g., BAX) determines cytochrome c release and cell fate [15–19]. Inhibitor of apoptosis proteins (IAPs), including X-linked IAP (XIAP) and Survivin, can directly bind and inhibit caspases to suppress cell death [20–23]. Additionally, cell-cycle regulators such as *CDKN1A*/p21 and *CDKN1B*/p27 integrate stress signals and modulate apoptotic susceptibility; both p21 and p27 have been implicated in the regulation of autophagy-apoptosis crosstalk [24–30].

Despite these advances, the apoptosis-related response of primary LSCs from patients with aniridia to an acute LPS challenge remains incompletely characterized. We hypothesized that AN-LSCs may respond differently from control donor-derived LSCs to standardized LPS stimulation. This study therefore compared apoptosis rates and selected apoptosis- and inflammation-related measures—including caspases, BCL-2 family members, inhibitor of apoptosis proteins, *CDKN1A*, *CDKN1B*, and TNF-α—in primary AN-LSCs and control LSCs following a standardized 24-hour LPS challenge.

## Materials and methods

### Ethics approval and consent to participate

This study was approved by the Ethics Committee of Saarland, Germany (No. 178/22), and conducted in accordance with the Declaration of Helsinki. Written informed consent was obtained from all participants. The recruitment period for this prospective study was from November 7, 2022, to December 15, 2024.

### Tissue acquisition and processing

Limbal specimens from eight patients with congenital aniridia (n = 8 eyes; mean age, 32.50 ± 17.07 years; range, 2–50 years) were obtained during clinically indicated ocular procedures at the Department of Ophthalmology, Saarland University Medical Center, Homburg/Saar, Germany. Preoperative staging of aniridia-associated keratopathy (AAK) was performed according to the criteria described by Lagali et al. [31,32]. Control specimens from eight donors (n = 8 donor eyes; mean donor age, 80.38 ± 4.56 years; range, 72–86 years) were obtained from donor corneoscleral tissue provided by the LIONS Cornea Bank Saar-Lor-Lux, Trier/Westpfalz, Homburg/Saar, Germany. Demographic characteristics, including sex distribution and relevant clinical parameters, are presented in Tables 1 and 2.

**Table 1:** Demographic characteristics of control corneal donors.

Group	n	Age, mean ± SD (range), years	Sex
Control donors	8	80.38 ± 4.56 (72–86)	5 (62.5%) Male; 3 (37.5%) Female

**Table 2:** Demographic characteristics and aniridia-associated keratopathy (AAK) grade of donors with congenital aniridia, according to the classification by Lagali et al. [31,32].

Group	n	Age, mean ± SD (range), years	Sex	AAK grade [31,32]
Donors with congenital aniridia	8	32.50 ± 17.07 (2-50)	5 (62.5%) Female; 3 (37.5%) Male	Grade 3 (25%); Grade 4 (75%)

### Primary cell Isolation

Aniridia limbal specimens were obtained during clinically indicated ocular procedures at Saarland University Medical Center, whereas control limbal specimens were obtained from donor corneoscleral tissue. Limbal specimens measuring approximately 1.5 × 1.5 mm from both groups were subsequently processed using the same standardized isolation protocol as previously described [33]. Briefly, specimens were enzymatically digested in KGM3 medium (PromoCell) supplemented with collagenase A (1 mg/mL; Roche, #10103578001) for 24 hours at 37 °C, gently dissociated mechanically, and passed through 40-µm Flowmi™ strainers (Bel-Art, #H13680-0040). The LSC-containing flow-through was collected for subsequent culture. Subsequent culture and passaging procedures were applied identically to control and aniridia-derived cell preparations.

### Cell-model characterization

The cell preparations used in the present study were not the same as those examined in our previous characterization study and thus were not directly phenotypically characterized. However, cells from both the control and aniridia groups were isolated, cultured, and passaged using the same standardized protocol. In our previous study, cell preparations generated using this protocol were characterized by assessment of PAX6 and stromal/keratocyte-associated markers, including COL1A1, COL3A1, COL5A1, ACTA2, ALDH3A1, KER, LUM, and CD34 [10]. No batch-specific phenotypic characterization was performed for the present experimental series; therefore, residual cellular heterogeneity and potential differences in cellular composition between the groups cannot be excluded.

### Cell culture establishment

The LSC-containing flow-through was collected and centrifuged at 1,500 × g for 5 minutes. The resulting cell pellets were resuspended in complete DMEM containing 5% FCS and seeded into 6-well culture plates. Cultures were maintained under standard conditions (37 °C, 5% CO_2_, 95% humidity), with medium changes every 48–72 hours until reaching approximately 90% confluence.

### LPS treatment

LSCs were cultured to approximately 80% confluence before experimental treatment. After a 24-hour stabilization period, the culture medium was replaced with DMEM containing lipopolysaccharide (LPS; Sigma-Aldrich®, Germany, #L2654) as a standardized acute innate inflammatory stimulus. Cells were exposed to LPS at final concentrations of 0 (untreated control), 2.5, 10, or 17.5 µg/mL for 24 hours [34].

### Apoptosis measurement

Cellular apoptosis was assessed using Annexin V-APC/propidium iodide (PI) dual staining (Invitrogen, #88–8007). Trypsinized cell suspensions were washed with binding buffer and incubated sequentially with 5 μL Annexin V-APC for 15 minutes at room temperature, followed by 5 μL PI. Fluorescence signals were acquired by flow cytometry using the designated channels for Annexin V-APC and PI. Cell populations were categorized as viable (Annexin V ^−^ /PI^−^), early apoptotic (Annexin V ⁺ /PI^−^), or late apoptotic/necrotic (Annexin V ⁺ /PI⁺) by quadrant analysis. For apoptosis analysis, cells were obtained from three independent control donors and three independent aniridia donors, with cells from each donor assessed under all four LPS conditions. Three technical replicate measurements within each donor and condition were averaged before inferential analysis. Donor, rather than the technical replicate or individual flow-cytometric event, was treated as the biological unit.

### RNA measurement

Total RNA was extracted using the NORGEN RNA Purification Plus Micro Kit, following the manufacturer’s protocol. RNA purity was confirmed spectrophotometrically (Analytik Jena), with A260/A280 ratios exceeding 1.8. For cDNA synthesis, 500 ng of total RNA was reverse transcribed using OneTaq RT-PCR reagents (New England Biolabs, NEB). All RNA and cDNA samples were stored at −80 °C until further analysis.

### qPCR

Gene expression analysis was performed using SYBR Green-based quantitative PCR assays (Vazyme), with primer assay information provided in Table 3. Each 10 μL reaction contained 1 μL cDNA, 1 μL Qiagen primer mix, 5 μL SYBR Green master mix, and 3 μL nuclease-free water. Amplification was carried out on a QuantStudio 5 Real-Time PCR System (Thermo Fisher Scientific) using the following thermal cycling conditions: 95 °C for 10 seconds, 60 °C for 30 seconds, and 72 °C for 15 seconds, repeated for 40 cycles. Relative gene expression levels were calculated using the 2^−ΔΔCt^ method, with β-glucuronidase (GUSB) serving as the internal reference gene.

**Table 3:** Primers used for qPCR.

Target cDNA	Qiagen Cat. No	Amplicon size	Transcript
Hs_CASP3_1_SG	QT00023947	147	NM_004346 (2689 bp)
Hs_CASP7_1_SG	QT00003549	149	NM_001227 (2607 bp)
Hs_CASP8_1_SG	QT00052416	61	NM_001080124 (2750 bp)
Hs_CASP9_1_SG	QT00036267	102	NM_001229 (3038 bp)
Hs_CASP10_1_SG	QT00002394	91	NM_001206542 (2178 bp)
Hs_BIRC5_1_SG	QT00081186	101	NM_001168 (2655 bp)
Hs_BAX_1_SG	QT00031192	111	NM_004324 (891 bp)
Hs_BID_1_SG	QT00077833	98	NM_001196 (2217 bp)
Hs_BCL2_1_SG	QT00025011	80	NM_000633 (6492 bp)
Hs_TNF_1_SG	QT00029162	98	NM_000594 (1686 bp)
Hs_XIAP_1_SG	QT00042854	87	NM_001167 (8460 bp)
Hs_GUSB_1_SG	QT00046046	96	NM_000181 (2321 bp)
Hs_CDKN1A_1_SG	QT00062090	79	NM_000389 (2175 bp)
Hs_CDKN1B_2_SG	QT00998445	146	NM_004064 (2535 bp)

### Flow cytometry

Following dissociation with non-EDTA trypsin, cells were fixed and permeabilized using a commercial fixation/permeabilization system according to the manufacturer’s instructions. Fixed and permeabilized cells were incubated with the primary antibodies listed in Table 4, followed by the corresponding fluorochrome-conjugated secondary antibodies. Secondary-antibody-only controls were processed in parallel to assess background fluorescence. Samples were analyzed using a CytoFLEX flow cytometer (Beckman Coulter) and CytExpert 2.6 software, and fluorescence values were expressed relative to the corresponding controls. No brefeldin A, monensin, or other protein-transport inhibitor was used before TNF-α staining. Consequently, the flow-cytometric TNF-α signal cannot be interpreted as a quantitative measure of total intracellular TNF-α production and is therefore reported as cell-associated TNF-α fluorescence under the experimental conditions. Secreted TNF-α was assessed independently in culture supernatants by ELISA.

**Table 4:** Antibodies used for flow cytometry.

Antibody	Dilution	Supplier	Catalog number	Source
TNF-α (D1G2) Rabbit mAb	1:100	Cell signaling	#8184S	Rabbit
Bid Polyclonal antibody	1:100	Proteintech	#10988–1-AP	Rabbit
Bax (E4U1V) Rabbit mAb	1:100	Cell signaling	#41162S	Rabbit
Human Bcl-2 Polyclonal antibody	1:100	Proteintech	#12789–1-AP	Rabbit
XIAP Polyclonal antibody	1:100	Proteintech	#23453–1-AP	Rabbit
Survivin (71G4B7) Rabbit mAb	1:100	Cell signaling	#2808S	Rabbit
p21 Waf1/Cip1 (12D1) Rabbit mAb	1:100	Cell Signaling	#2947	Rabbit
p27 Kip1 (SX53G8.5) Mouse mAb	1:100	Cell Signaling	#3698	Mouse
Caspase-9 Antibody	1:100	Cell Signaling	#9502	Rabbit
Caspase-3 Antibody	1:100	Proteintech	#19677–1-AP	Rabbit
Cleaved Caspase-7 (Asp198) (D6H1) Rabbit mAb	1:100	Cell signaling	#8438S	Rabbit
Cleaved Caspase-8 (Asp374) (E6H8S) Rabbit mAb	1:100	Cell signaling	#98134S	Rabbit
Caspase-10 Polyclonal antibody	1:100	Proteintech	#14311–1-AP	Rabbit
Goat anti-Rabbit IgG (Heavy & Light Chain) Antibody (FITC) – Preadsorbed	1:500	Antibody online	ABIN101988	–
IgG1 Antibody, anti-mouse, REAfinity	1:50	Miltenyi Biotec	F0118	–

### ELISA

Secreted TNF-α levels were quantified using the DuoSet ELISA kit (R&D Systems, #DY210), following the manufacturer’s protocol. Absorbance was measured at 450 nm using a Tecan F50 microplate reader. Cytokine concentrations were normalized to total protein content, as determined by a bicinchoninic acid (BCA) assay.

### Statistical analysis

qPCR data were analyzed on log2-transformed 2^−ΔΔCt^ fold-change values using two-way mixed-effects models fitted by restricted maximum likelihood (REML), because some repeated observations were missing. Group was treated as the between-donor factor, LPS condition as the within-donor repeated factor, and the Group × LPS interaction was included. Flow-cytometry and ELISA outcomes were analyzed using two-way repeated-measures ANOVA with the same factor structure. The Geisser–Greenhouse correction was applied to repeated-measures omnibus tests. For qPCR, flow-cytometry, and ELISA outcomes, prespecified within-group comparisons of each LPS-treated condition with the corresponding untreated condition were adjusted using Dunnett’s procedure, and prespecified between-group comparisons at each LPS concentration were adjusted using the Šídák method. The qPCR, flow-cytometry, and ELISA analyses were performed using GraphPad Prism 11.0.0.93. qPCR graphs display the untransformed 2^−ΔΔCt^ fold-change values, whereas flow-cytometry and ELISA data are displayed as arithmetic means ± SD.

Apoptosis rates were analyzed using an exploratory linear mixed-effects model. Three technical replicates were averaged within each donor and condition before inferential analysis, and donor was treated as the biological unit. The model included group, categorical LPS condition, the Group × LPS interaction, and donor age. Donor was treated as the repeated-measures subject, with restricted maximum likelihood (REML) estimation and a compound-symmetry covariance structure. Omnibus fixed effects were evaluated using F tests. Following the significant Group × LPS interaction, prespecified within-group comparisons of each LPS-treated condition with the corresponding untreated condition and between-group comparisons of the change from the untreated condition were adjusted using Dunnett’s procedure. The mixed-effects analysis was performed using custom Python code (Python 3.12.13; Python Software Foundation), implemented with NumPy 2.3.5, pandas 2.2.3, and SciPy 1.17.0.

No formal a priori power calculation was performed because the sample size was constrained by the availability of primary human tissue from this rare disease population. Given the small number of independent donors, formal normality testing would have limited ability to detect departures from normality and was therefore not considered sufficient to establish the distributional assumption. Accordingly, all inferential analyses were considered exploratory and should be interpreted cautiously, with confirmation in a larger independent cohort of appropriately age-matched donors required.

## Results

### Apoptosis rate

At baseline, the mean apoptosis rate was numerically higher in AN-LSCs than in control LSCs (2.10% vs. 0.52%); however, this between-group difference was not statistically significant at the donor level. The exploratory linear mixed-effects model revealed a significant main effect of LPS condition (F(3,12) = 52.037, P < 0.001) and a significant Group × LPS interaction (F(3,12) = 7.475, P = 0.004). Neither the group main effect (F(1,3) = 0.328, P = 0.607) nor donor age (F(1,3) = 0.553, P = 0.511) reached statistical significance. Dunnett-adjusted within-group comparisons showed significant increases in apoptosis at 10 and 17.5 µg/mL LPS relative to the corresponding untreated condition in control LSCs (adjusted P = 0.0177 and P = 0.0009, respectively) and AN-LSCs (adjusted P = 0.0009 and P < 0.0001, respectively). The changes at 2.5 µg/mL were not statistically significant in either group. The increase in apoptosis from the untreated condition to 17.5 µg/mL LPS was significantly greater in AN-LSCs than in control LSCs (adjusted P = 0.003), whereas the between-group change contrasts at 2.5 and 10 µg/mL were not significant (Fig 1).

**Fig 1:**
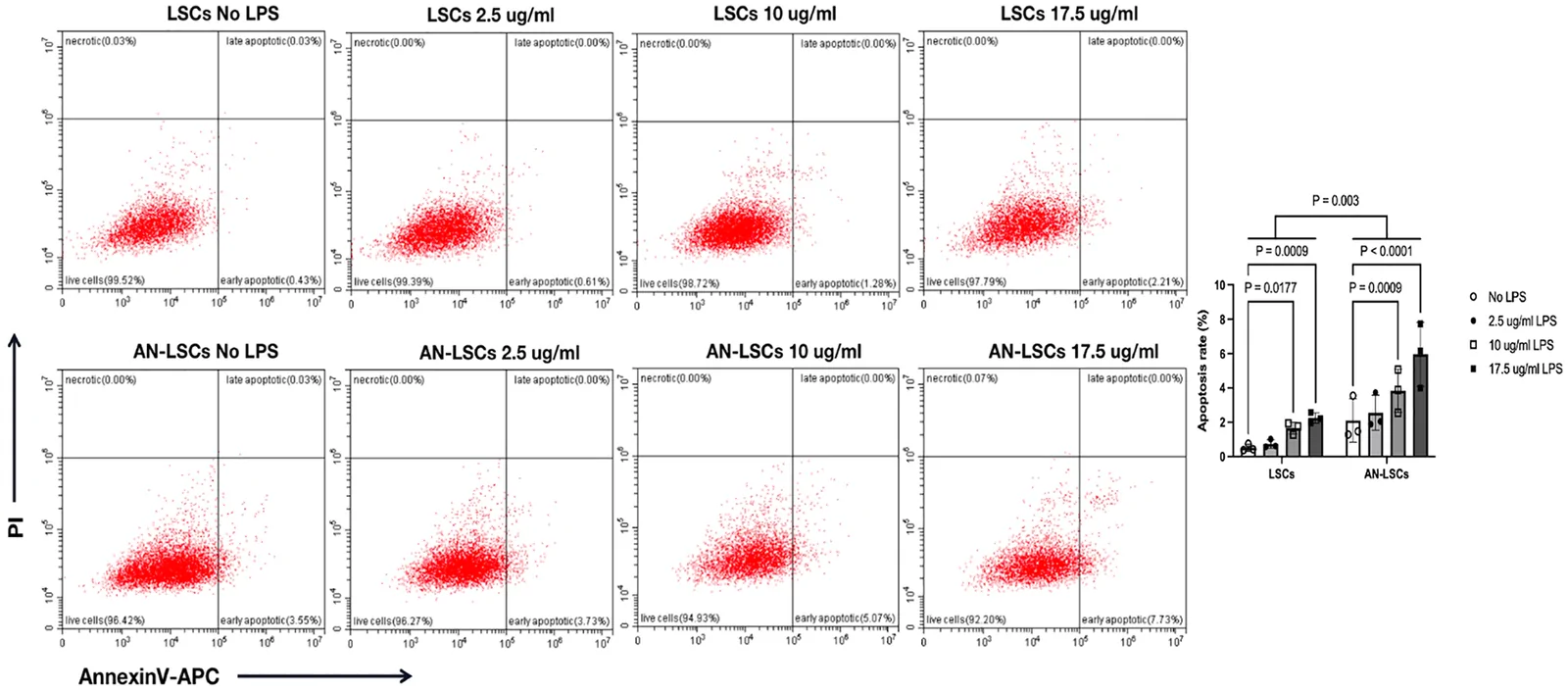
Apoptosis rates of control limbal stromal cells (LSCs) and aniridia-derived LSCs (AN-LSCs) after acute LPS exposure. Cells were exposed to 0, 2.5, 10, or 17.5 µg/mL LPS for 24 h and analyzed by Annexin V-APC/PI flow cytometry. Representative flow-cytometry plots and mean ± SD values from three independent donors per group are shown. Three technical replicates were averaged within each donor and condition before inferential analysis. Statistical analysis used an exploratory linear mixed-effects model with donor as the repeated-measures subject. Dunnett-adjusted within-group comparisons showed significant increases in apoptosis at 10 and 17.5 µg/mL LPS compared with the corresponding untreated condition in control LSCs (adjusted P = 0.0177 and 0.0009, respectively) and AN-LSCs (adjusted P = 0.0009 and < 0.0001, respectively). Changes at 2.5 µg/mL were not statistically significant. The upper comparison indicates the Dunnett-adjusted between-group contrast in the change from untreated to 17.5 µg/mL LPS (adjusted P = 0.003); the corresponding between-group change contrasts at 2.5 and 10 µg/mL were not significant.

### mRNA levels of apoptosis-related markers

Analysis of the log2-transformed qPCR data showed that no Group × LPS interaction reached statistical significance for any of the 13 markers examined (all P > 0.05). Šídák-adjusted between-group comparisons were also nonsignificant at all concentrations, except for higher *CDKN1A* mRNA levels in AN-LSCs than in control LSCs at 2.5 µg/mL LPS (adjusted P = 0.0085). In control LSCs, Dunnett-adjusted increases relative to the untreated condition were detected for *BID* at 2.5, 10, and 17.5 µg/mL (P = 0.0195, 0.0065, and 0.0068), *CASP7* at 10 and 17.5 µg/mL (P = 0.0214 and 0.0044), *CDKN1B* at 10 and 17.5 µg/mL (P = 0.0005 and 0.0010), *XIAP* at all three concentrations (P ≤ 0.0006), and *CASP10* at 10 µg/mL (P = 0.0468).

In AN-LSCs, Dunnett-adjusted increases relative to the untreated condition were detected for *BID* at 2.5 and 10 µg/mL (P = 0.0451 and 0.0131), *BAX* at 17.5 µg/mL (P = 0.0100), *CASP3* at 10 and 17.5 µg/mL (P = 0.0239 and 0.0027), *CASP7* at 10 and 17.5 µg/mL (P = 0.0165 and 0.0024), *CDKN1B* at all three concentrations (P ≤ 0.0006), *TNF* at 17.5 µg/mL (P = 0.0034), and *XIAP* at all three concentrations (P ≤ 0.0021). No significant within-group comparison was detected for *BCL2*, *BIRC5*, *CASP8*, or *CASP9* (Fig 2).

**Fig 2:**
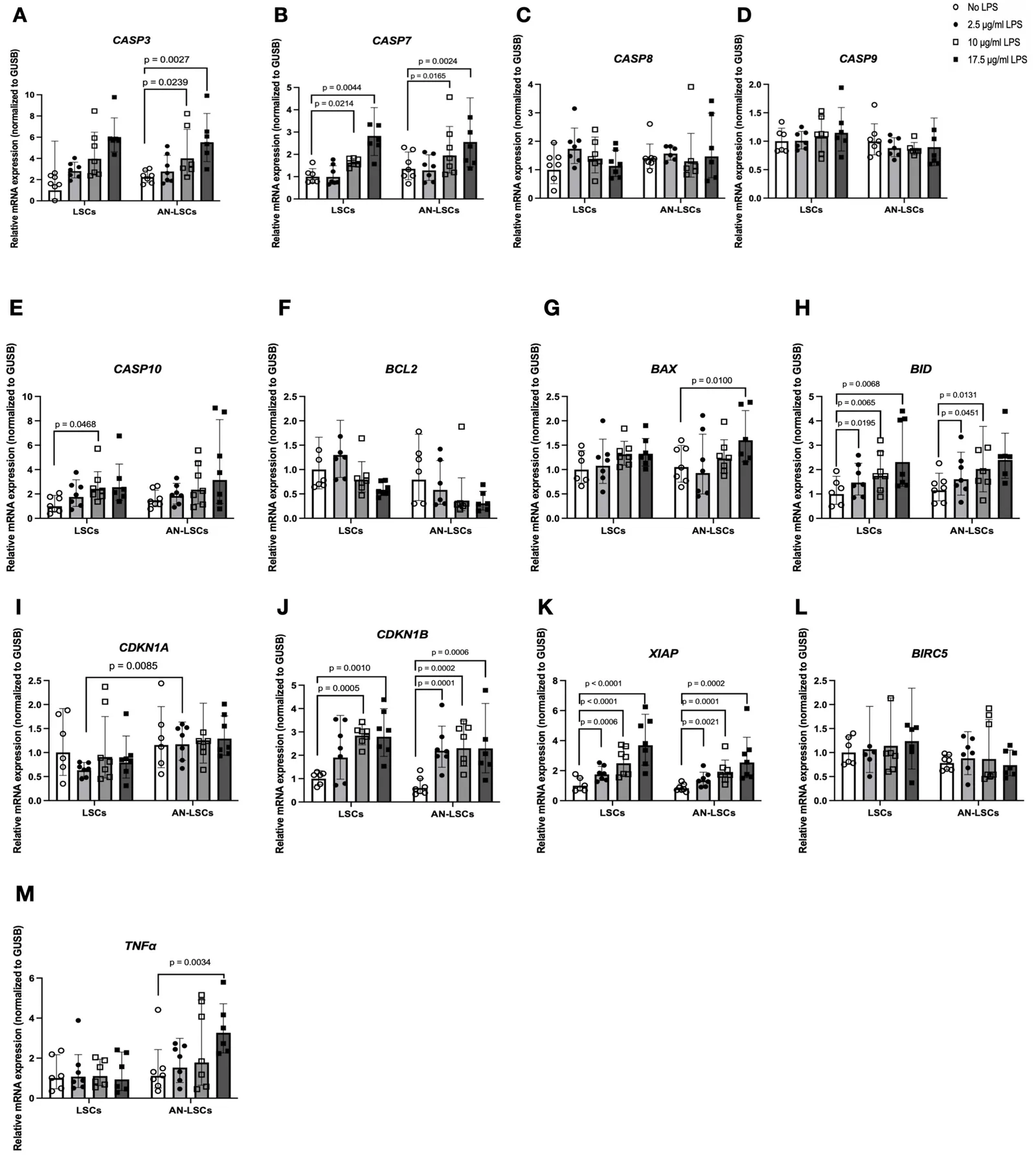
mRNA levels of apoptosis-related markers in control limbal stromal cells (LSCs) and aniridia-derived LSCs (AN-LSCs) following acute LPS exposure. *CASP* family genes (A–E), *BCL2* family genes (F–H), and other apoptosis-related genes (I–M) were analyzed by qPCR following exposure to 0, 2.5, 10, or 17.5 µg/mL LPS for 24 h. Graphs show untransformed 2^−ΔΔCt^ fold-change values as mean ± SD from up to seven independent donors per group; missing observations were treated as missing. Statistical analyses were performed on log2-transformed fold-change values using two-way mixed-effects models fitted by restricted maximum likelihood (REML), with group as the between-donor factor, LPS condition as the within-donor repeated factor, and the Group × LPS interaction included. Displayed within-group comparisons of each LPS-treated condition with the corresponding untreated condition were Dunnett-adjusted, and between-group comparisons at each LPS concentration were Šídák-adjusted. Only statistically significant adjusted comparisons are displayed.

### Protein levels of apoptosis-related markers

Flow-cytometry analyses showed that no Group × LPS interaction reached statistical significance and that no Šídák-adjusted between-group comparison was significant for any marker (all adjusted P > 0.05). Among the caspase-family markers, Caspase-10-associated fluorescence increased in control LSCs at 17.5 µg/mL LPS compared with the untreated condition (Dunnett-adjusted P = 0.0197); no other within-group caspase comparison was significant (Fig 3). For the Bcl-2-family markers, Bcl-2-associated fluorescence decreased in control LSCs at 10 µg/mL LPS (Dunnett-adjusted P = 0.0331), and Bax-associated fluorescence decreased in AN-LSCs at 2.5 µg/mL LPS (Dunnett-adjusted P = 0.0434); no other within-group comparison was significant (Fig 4).

**Fig 3:**
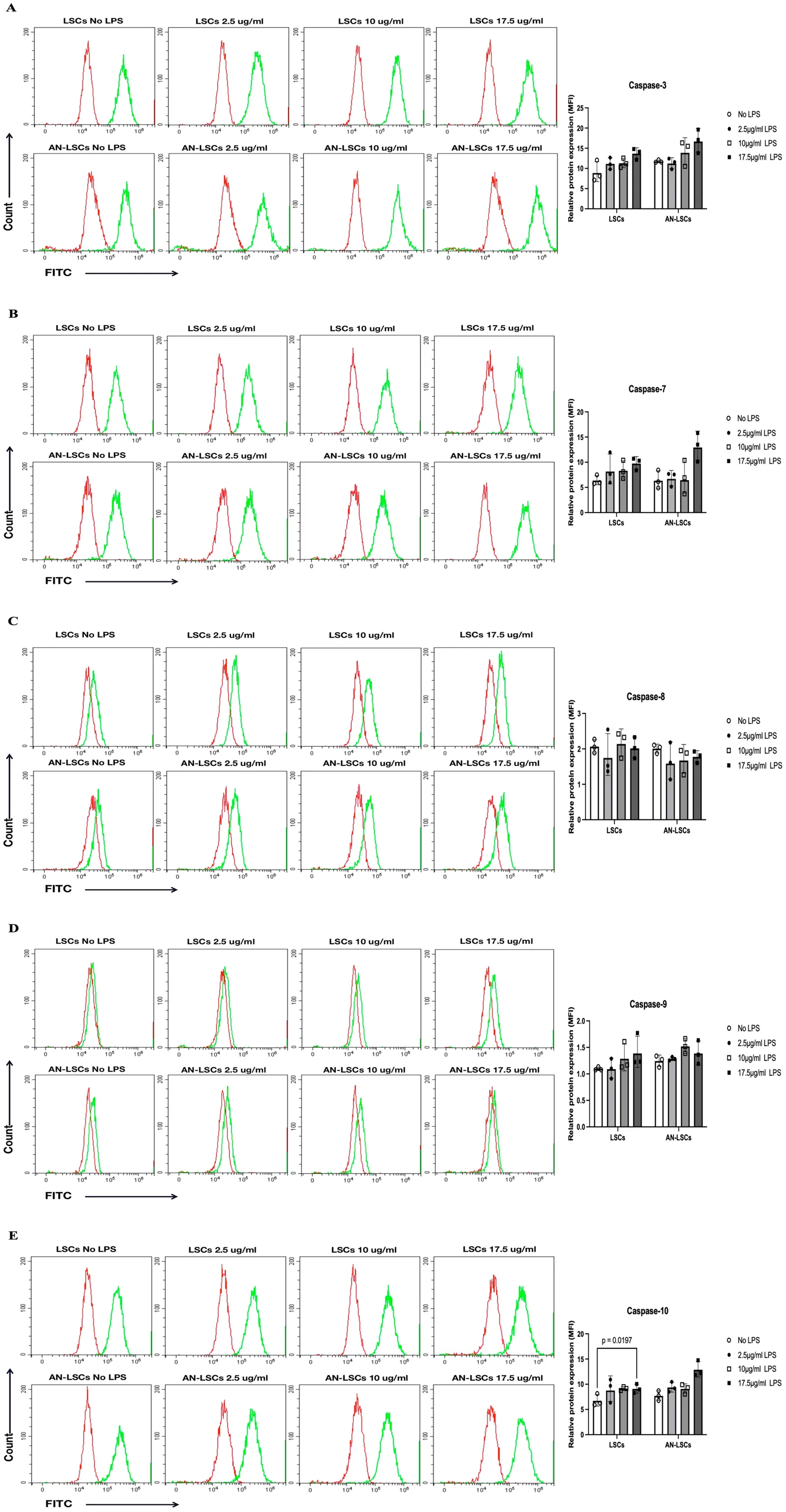
Protein levels of caspase-family markers in control limbal stromal cells (LSCs) and aniridia-derived LSCs (AN-LSCs) after acute LPS exposure, assessed by flow cytometry. Caspase-3, −7, −8, −9, and −10 were assessed by flow cytometry (A–E) following exposure to 0, 2.5, 10, or 17.5 µg/mL LPS for 24 h. Green curves represent primary-antibody staining followed by the corresponding fluorochrome-conjugated secondary antibodies, whereas red curves represent secondary-antibody-only controls. Normalized mean fluorescence intensity (MFI) ratios are presented as mean ± SD from three independent donors per group. Data were analyzed using two-way repeated-measures ANOVA with Geisser-Greenhouse correction. Displayed P values represent Dunnett-adjusted within-group comparisons of each LPS-treated condition with the corresponding untreated condition. Between-group comparisons at each LPS concentration were Šídák-adjusted and are not displayed because none reached statistical significance.

**Fig 4:**
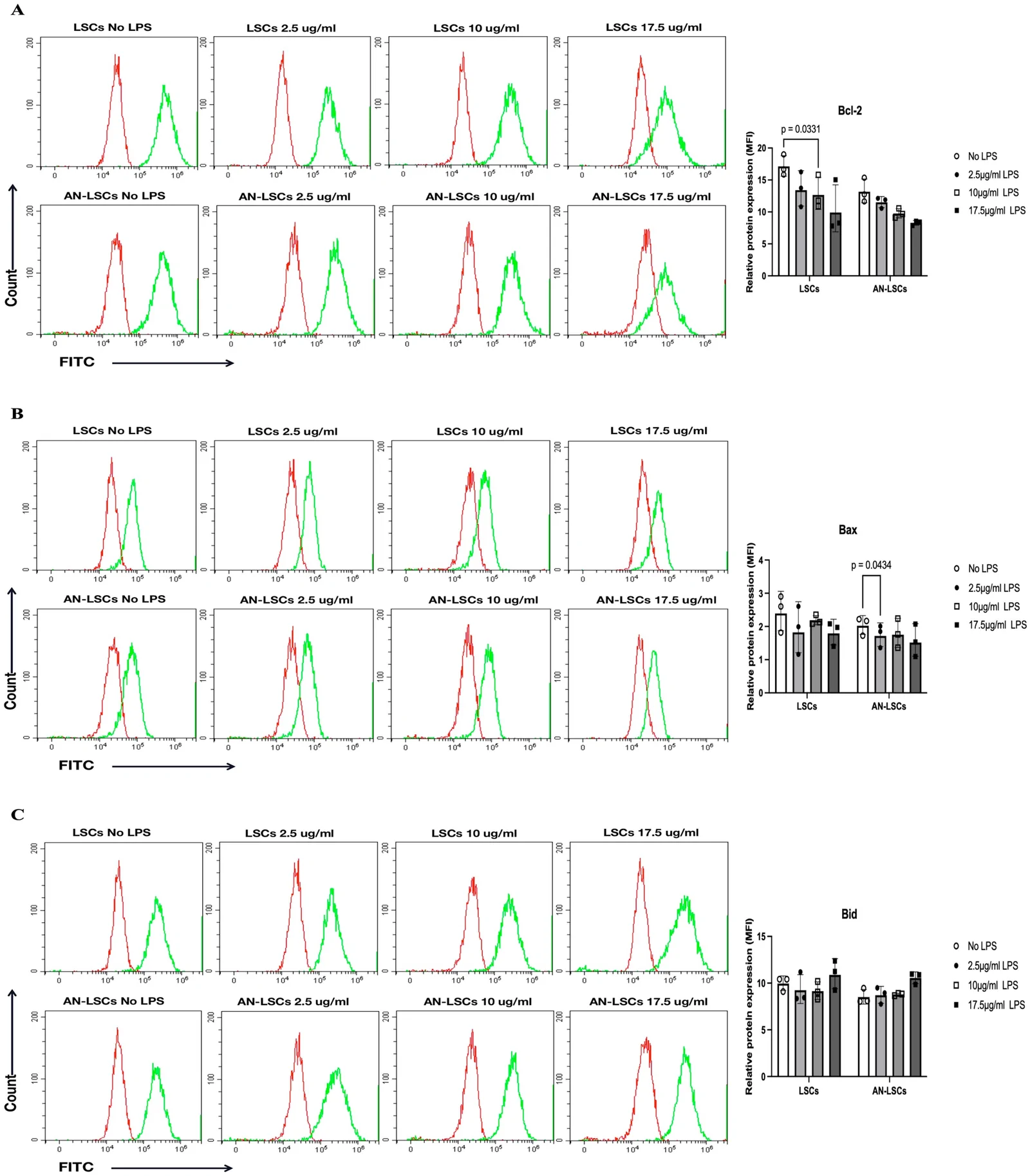
Protein levels of Bcl-2-family markers in control limbal stromal cells (LSCs) and aniridia-derived LSCs (AN-LSCs) after acute LPS exposure, assessed by flow cytometry. Bcl-2, Bax, and Bid were assessed by flow cytometry (A–C) following exposure to 0, 2.5, 10, or 17.5 µg/mL LPS for 24 h. Green curves represent primary-antibody staining followed by the corresponding fluorochrome-conjugated secondary antibodies, whereas red curves represent secondary-antibody-only controls. Normalized mean fluorescence intensity (MFI) ratios are presented as mean ± SD from three independent donors per group. Data were analyzed using two-way repeated-measures ANOVA with Geisser–Greenhouse correction. Displayed P values represent Dunnett-adjusted within-group comparisons of each LPS-treated condition with the corresponding untreated condition. Between-group comparisons at each LPS concentration were Šídák-adjusted and are not displayed because none reached statistical significance.

For the flow-cytometric measures in Fig 5, XIAP-associated fluorescence increased in AN-LSCs at 17.5 µg/mL LPS compared with the untreated condition (Dunnett-adjusted P = 0.0295), whereas cell-associated TNF-α fluorescence decreased at 17.5 µg/mL LPS (Dunnett-adjusted P = 0.0485). No other within-group comparison for p21, p27, XIAP, Survivin, or cell-associated TNF-α fluorescence was significant. Secreted TNF-α levels measured by ELISA showed a significant overall group effect, with higher values in AN-LSCs than in control LSCs across LPS conditions (P = 0.0087). In AN-LSCs, secreted TNF-α levels increased at 17.5 µg/mL LPS compared with the untreated condition (Dunnett-adjusted P = 0.0348), whereas no significant within-group change was observed in control LSCs. However, no between-group comparison at **any** individual LPS concentration was significant after Šídák adjustment (Fig 5).

**Fig 5:**
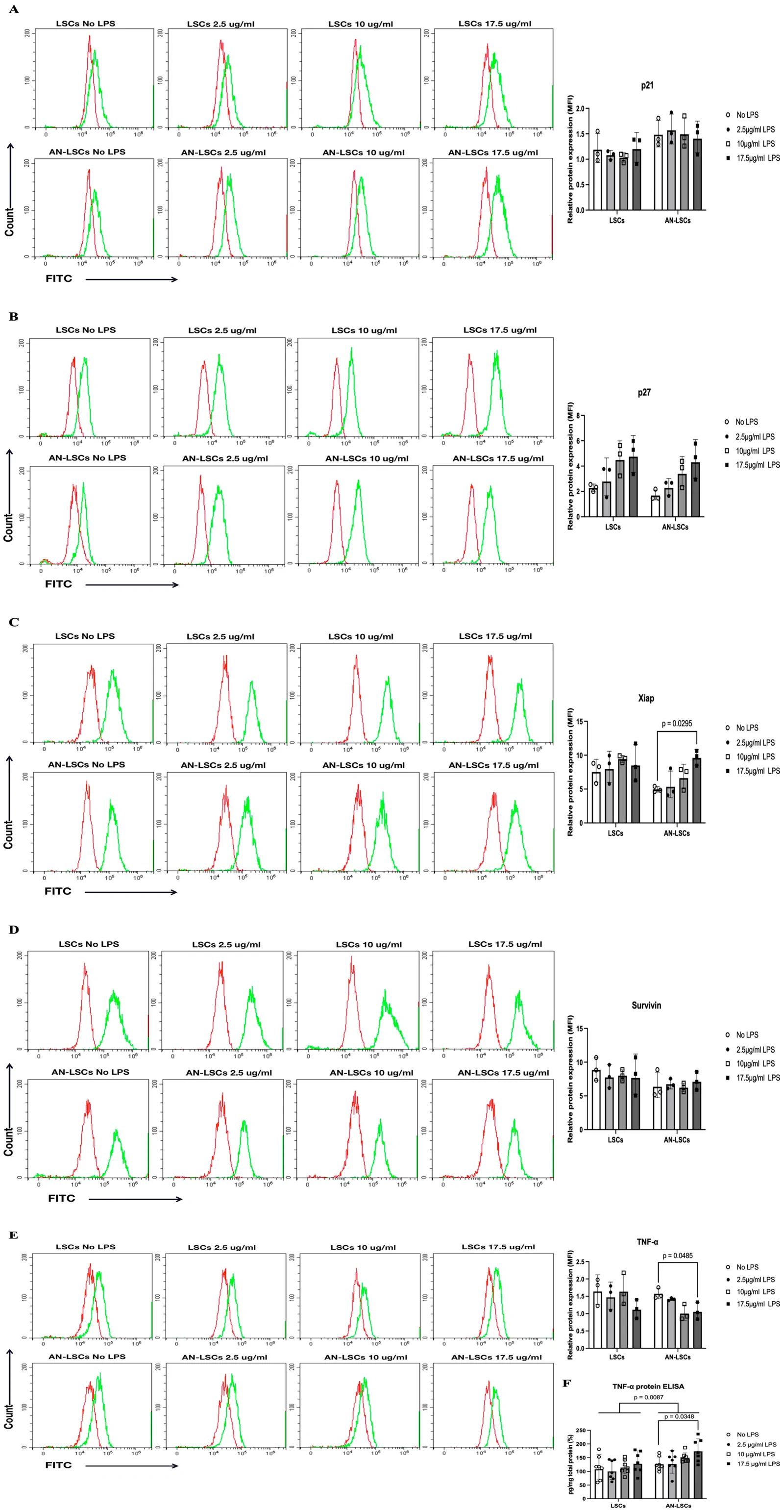
Apoptosis- and inflammation-related protein measures in control limbal stromal cells (LSCs) and aniridia-derived LSCs (AN-LSCs) following acute LPS exposure. p21, p27, XIAP, Survivin, and cell-associated TNF-α fluorescence were assessed by flow cytometry (A–E), whereas secreted TNF-α was measured in culture supernatants by ELISA (F), following exposure to 0, 2.5, 10, or 17.5 µg/mL LPS for 24 h. Flow-cytometry data are presented as mean ± SD from three independent donors per group and were analyzed using two-way repeated-measures ANOVA with the Geisser–Greenhouse correction. Displayed flow-cytometry P values represent Dunnett-adjusted within-group comparisons of each LPS-treated condition with the corresponding untreated condition. Between-group flow-cytometry comparisons at each LPS concentration were Šídák-adjusted and are not displayed because none reached statistical significance. ELISA data are presented as mean ± SD from seven independent donors per group and were analyzed using two-way repeated-measures ANOVA with the Geisser–Greenhouse correction. For ELISA, prespecified within-group comparisons of each LPS-treated condition with the corresponding untreated condition were Dunnett-adjusted, and between-group comparisons at each LPS concentration were Šídák-adjusted. Only statistically significant effects and adjusted comparisons are displayed.

## Discussion

This study identified LPS-associated changes in selected apoptosis- and inflammation-related measures in control LSCs and AN-LSCs. The mean basal apoptosis rate was numerically higher in AN-LSCs, although the donor-level difference was not statistically significant. In the molecular analyses, most adjusted between-group comparisons were nonsignificant: the only significant qPCR comparison was higher *CDKN1A* mRNA in AN-LSCs at 2.5 µg/mL LPS, and no adjusted flow-cytometric between-group comparison was significant. Accordingly, the data do not support broad baseline differences in the examined molecular markers.

The acute LPS challenge increased apoptosis in both groups, with a greater increase in AN-LSCs at 17.5 µg/mL. In contrast, no Group × LPS interaction reached statistical significance in the qPCR or flow-cytometry analyses. Marker-specific within-group changes therefore should not be interpreted as evidence that the molecular response to LPS differed significantly between AN-LSCs and control LSCs. Flow cytometry also detected a reduction in cell-associated TNF-α fluorescence in AN-LSCs at 17.5 µg/mL, whereas secreted TNF-α measured by ELISA showed a significant overall group effect, with higher levels in AN-LSCs across LPS conditions, as well as a significant increase at 17.5 µg/mL LPS compared with untreated AN-LSCs. Because no protein-transport inhibitor was used before TNF-α staining, the flow-cytometric signal cannot be interpreted as a quantitative measure of total intracellular TNF-α production, but rather reflects cell-associated TNF-α fluorescence under the experimental conditions.

The transcript- and protein-level findings were not uniformly concordant. For example, *BAX* mRNA increased in AN-LSCs at 17.5 µg/mL LPS, whereas Bax-associated fluorescence decreased at 2.5 µg/mL. The absence of significant *CASP9* changes, together with marker-specific changes in other caspase-related measures, does not permit the relative contributions of mitochondrial and extrinsic pathways to be determined without direct functional assays [16–19]. Similarly, the observed *TNF*-α and caspase-associated changes are compatible with potential relationships between inflammatory and apoptotic responses, but no causal interaction or feedback loop was directly tested [12,13,18,19].

Fig 6 presents a hypothesis-generating working model focused on LPS-associated marker changes in AN-LSCs. The schematic does not imply that molecular responses to LPS differed significantly between AN-LSCs and control LSCs, because no molecular Group × LPS interaction reached statistical significance. Possible relationships involving inflammatory signaling, TNF-α, mitochondrial regulation, and caspase activation are presented as hypotheses derived from prior literature rather than as pathways directly demonstrated in the present study [12,13,16–19,22,23,27,29,30,35–37].

**Fig 6:**
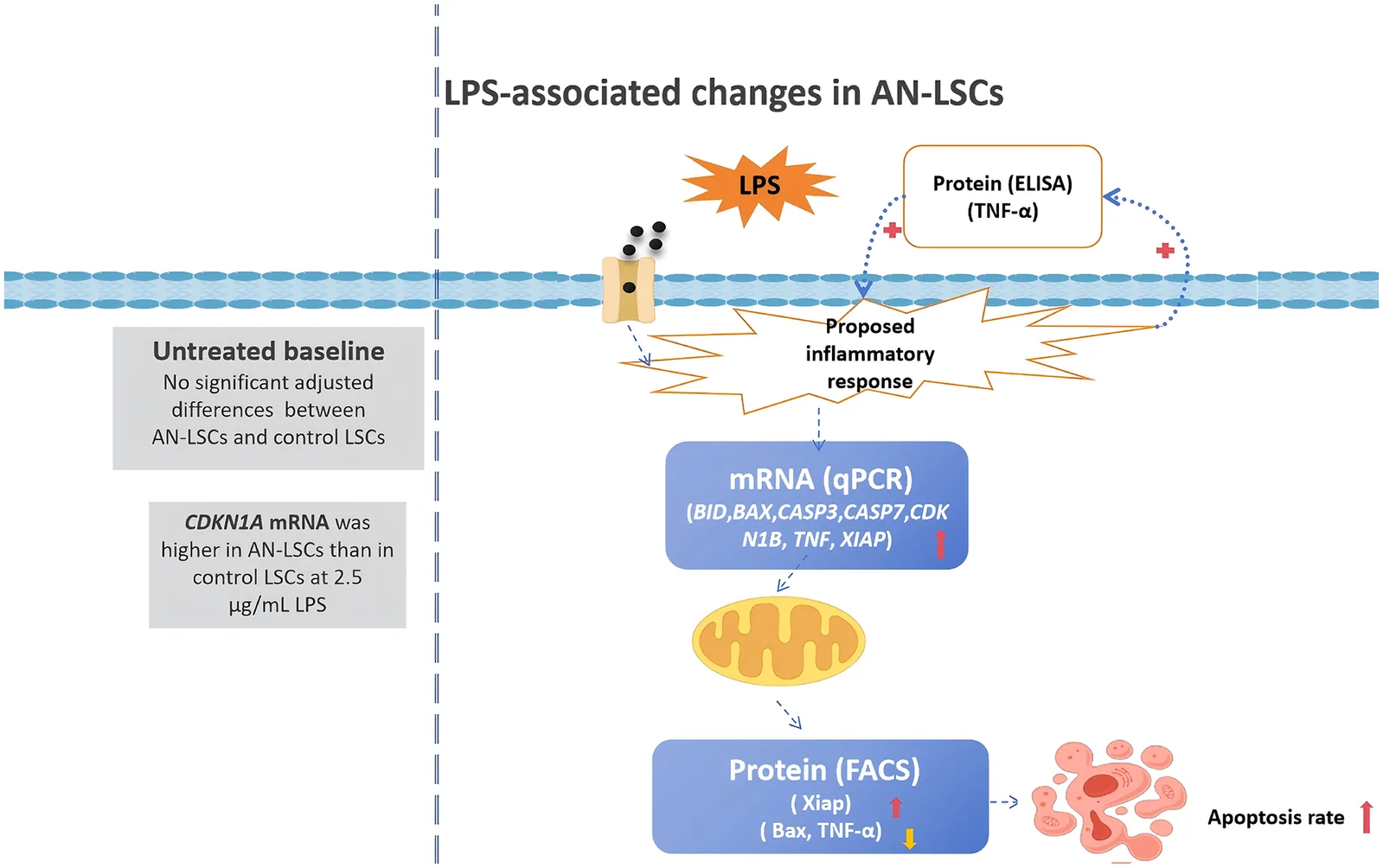
Hypothesis-generating working model of LPS-associated marker changes in primary human aniridia-derived limbal stromal cells (AN-LSCs). Note: This is a hypothesis-generating model; the relationships depicted have not been causally established by the present data. No significant adjusted baseline differences in the examined qPCR or flow-cytometry markers were detected between untreated AN-LSCs and control LSCs. At 2.5 µg/mL LPS, *CDKN1A* mRNA was higher in AN-LSCs than in control LSCs. At selected LPS concentrations, *BID*, *BAX*, *CASP3*, *CASP7*, *CDKN1B*, TNF, and *XIAP* mRNA levels increased within AN-LSCs relative to the untreated condition. XIAP-associated fluorescence increased, whereas Bax- and cell-associated TNF-α fluorescence decreased within AN-LSCs relative to the untreated condition. Secreted TNF-α levels and apoptosis rates increased following LPS exposure in AN-LSCs. No molecular Group × LPS interaction reached statistical significance. Dashed arrows and the dotted TNF-α loop indicate hypothetical, untested relationships and should not be interpreted as causal pathways.

The observed marker patterns may help generate hypotheses for future mechanistic and translational studies. Larger, appropriately age-matched independent donor cohorts and functional experiments will be required before any of these markers can be considered potential clinical biomarkers or therapeutic targets [23,29,35,36]. Evaluation of pathway-specific inhibitors may be informative in future studies; however, the present data neither establish treatment efficacy nor support clinical recommendations [23,38].

This study has several important limitations. Although primary limbal tissue was available from eight donors per group, the apoptosis analysis included only three independent donors per group, substantially limiting statistical power and generalizability. The markedly different and non-overlapping age distributions prevent disease-associated effects from being reliably separated from age-associated effects; a nonsignificant age effect in the exploratory mixed-effects model does not exclude residual age-related confounding. The specific cell preparations used here did not undergo batch-specific phenotypic characterization; therefore, residual cellular heterogeneity and potential differences in cellular composition cannot be excluded. In addition, the absence of pathway-specific functional validation limits causal inference, and the 24-hour LPS challenge does not reproduce the complex chronic inflammatory microenvironment of AAK. The findings should therefore be interpreted as responses to a defined acute LPS stimulus and should not be generalized to other inflammatory stimuli or to chronic AAK.

Future studies should include larger, appropriately age-matched independent donor cohorts, batch-specific phenotypic characterization of the cell preparations, and pathway-focused functional assays. Complementary approaches, such as single-cell transcriptomics and metabolic analyses, may help resolve cellular heterogeneity and identify candidate mechanisms for direct experimental testing.

In summary, the present data show a significant Group × LPS interaction for apoptosis, with a greater increase in AN-LSCs at 17.5 µg/mL, together with marker- and concentration-specific molecular changes. No Group × LPS interaction reached statistical significance in the qPCR or flow-cytometry analyses. Given the small number of independent donors, the marked donor-age imbalance, and other methodological limitations, these findings should be considered exploratory and hypothesis-generating and require confirmation in larger independent cohorts of appropriately age-matched donors.

## Conclusions

In this study, acute LPS exposure increased apoptosis in both control LSCs and AN-LSCs at 10 and 17.5 µg/mL, with a significantly greater increase in AN-LSCs at the highest concentration. The mean basal apoptosis rate was numerically higher in AN-LSCs, although the donor-level baseline difference was not statistically significant. qPCR and flow-cytometry analyses identified marker-specific LPS-associated changes, but no molecular Group × LPS interaction reached statistical significance.

The significant Group × LPS interaction for apoptosis indicates that the response pattern across LPS conditions differed between AN-LSCs and control LSCs; however, the present findings do not establish a causal relationship between inflammatory and apoptotic responses or support the existence of an inflammation–apoptosis feedback loop.

Given the small number of independent donors, the markedly different and non-overlapping age distributions between groups, the absence of batch-specific phenotypic characterization, and the acute nature of the experimental model, these findings should be considered preliminary, exploratory, and hypothesis-generating and require validation in a larger independent cohort of appropriately age-matched donors before clinical or therapeutic implications can be drawn.

## Supporting information

S1 Data Anonymized donor-level numerical data underlying Figures 1–5. (XLSX)
